# Annotations

**Published:** 1890-03-01

**Authors:** 


### Annotations.

"Imitation is the sincerest form of flattery." The show of dressed dolls initiated by this journal a few months ago was generally appreciated on account of its novelty and universal interest. The New Hospital for Women, it is said, is going to make a similar show one of the chief attractions of a bazaar to be held in April. We are glad that our idea is so soon bearing fruit. Hospital life, in so far as the officials are concerned, is by no means of the brightest. For one thing it is entirely unrelieved by the natural pleasures of home. The very shape and construction of the building suggests discipline, and nothing but discipline. Every hour of the day is mapped out by rule and line. Even relaxation is taken according to order, and always at the same time. It is no wonder, therefore, that anything which breaks the monotony or carries away the mind to outside excitement is eagerly welcomed. The dressed dolls which attracted so many visitors to the Charing Cross Hospital, expressed in very picturesque fashion the feelings and mental attitudes of those who fashioned them. Some of the dolls were very small to begin with, carelessly and even dowdily dressed, plainly showing that those who had dressed them had little pride in their calling, and small sense of its dignity and honour. Others, again, showed that they were the work of women who had born aptitudes for neatness, order, cleanliness, and perfectness in everything they did. A few were decidedly graceful, and had that light and airy look which proved that the brain which evolved them, though that of a sick nurse, was also that of a woman who had other ideas than those of nursing. The dressing of dolls, which is very interesting in itself, is only one of many bright and amusing relaxations which hospital nurses might find pleasure in. Now that the idea of relieving the tedium of hospital life has been fairly started and accepted, we cannot but express the hope that many fertile brains, both inside and outside hospitals, will set themselves to work to devise new recreations, and so to make nurses both happier as women and more efficient as attendants upon the sick.

**Dressed Dolls.**

Medical men are becoming a little shy of the bacillus and his legions of relatives. It was high time. One might have thought that humoralists and solidists in Pathology and Phlebotomists in practice had never been heard of by the eager young medical scientists of the day. Many of their older *confreres*, too, and some of them of no mean eminence, have swallowed with greedy appetite the crudest theories that the rawest German experimenters have seen fit to propound. But homely common sense seems to be reviving in the medical mind in this country; and even such high authorities as German and English house surgeons are asked to produce some little show of evidence in proof of the validity of their latest discoveries. Cancer, we were assured long ago, was a bicillary disease; but the *Medical Annual* for the current year speaks with quite refreshing caution on the subject. "Several endeavours," it reports, "have been made to prove the cause of cancer to be a bacillus. Kübasoff, after prolonged investigations, says cancer is caused by a special rod-shaped microbe. The bacilli have slightly ovoid outlines and are arranged mostly in pairs." Happy bacilli! "Their length amounts to one-fourth the diameter of a red-blood corpuscle. The bacillus can be cultivated, and when inoculated under the skin of animals, gives rise to a cancerous degeneration." The evidence would have been more convincing if the experimentor had said that the inoculations give rise to living cancerous growths. Degenerations are dangerous things to play with. He does, indeed, say that these degenerations commence "in the nearest lymphatic glands, and then generalise, forming genuine cancerous nodules. All varieties of cancer appear to be formed by the same bacillus." That is as if the same seed should give rise to a soft pulpy turnip and to the scirrhous roots of an ash tree. Well may the *Annual* say that "the foregoing must be accepted with great caution. It is most difficult to understand how one form of bacillus can give rise to all the clinical varieties of cancer and their different microscopic characters." We have often in this journal entered protests against the unscientific eagerness with which new discoveries are accepted, and the still more uncritical mental feebleness which makes so many men believe that what is new must necessarily also be true. If medical men would save their credit as pioneers in the march of science, they must demand evidence for every discovery that is announced, and evidence which will appeal to and convince the plain and unsophisticated mind. Science has always derided mysticism in theology and transcendentalism in metaphysics. Is the scientific medicine of modern times to be degraded into a hierarchy of priestly discoverers on the one hand, and on the other a mere uncultured laity of ordinary doctors who receive the oracular utterances of the priestly class without evidence and without dispute? The bare idea is ridiculous! Most medical men have to devote themselves to clinical work; some few live in the laboratory. The conclusions of the laboratory should constitute the basis of bedside practice. But what clinical worker who has the least self respect will found any of his practice on laboratory results which furnish no adequate evidence either of their scientific validity or of their practical worth?

**A Bacillary Check.**

Although Mr. Thiselton Dyer's book on the "Loves and Marriages of Some Eminent Men" has not been excessively praised by the critics, we have no doubt it will be found both soothing and consolatory by large numbers of men who do not belong to the critical class. Mr. Dyer gives such a long list of names of eminent persons who have married foolishly, and whose married lives have consequently proved entire failures, that one wonders whether he could match it with an equally extended list of marriages that have been entirely successful. Among the famous ones whose wives have been unworthy of their high destinies, Mr. Dyer mentions emperors, kings, statesmen, lawyers, poets, and all classes of persons who have claims to distinction of any kind. Some of the greatest and most illustrious of these have married downright termagants, others have selected the most common-place custodians of the keys and the plate basket, and some mere fools. The *Spectator* is much exercised concerning the want of sense and judgment displayed by the eminent ones. But the mystery is not after all so great. The fact of marriages being commonly made in youth explains a good deal of it. It is quite as true of able men as of stupid ones, that "you cannot put old heads on young shoulders." The very fact that a man is "able" is sometimes the explanation of his foolish marriage. For what is it that in course of time carries a man through the performance of deeds and achievements to a position of acknowledged eminence? Is it not courage and will quite as much as sense and judgment? But courage and will are endowments that not only develop early, but are born with a man. They are often, indeed, stronger at twenty than at forty. Sense and judgment, on the other hand, develop slowly, and are the products of middle and old age. If, therefore, an "able" young man takes a sudden and violent fancy for a young woman, his very ability at that period of life is all the more likely to make him marry her in defiance of the remonstrances of people who think themselves wiser than he. Besides, what man, especially what clever man, ever thinks of choosing a woman on account of her mere mental capacity? He chooses her because he is pleased with her, altogether without reference to her intellectual strength. Most commonly indeed, there is some mutual attraction between a man and a woman of a kind that neither of them cares to analyse, but which proves to be of sufficient strength and endurance to carry them up to and beyond the irrevocable marriage ceremony. On the whole it seems best that able men should sometimes marry termagants and fools, and should be blessed with children of a common-place order. They are thus kept within sympathetic range of the average man and woman. The strong brain and resolute will are apt to be accompanied by wings of vanity and self-confidence; and it is surprising to what heights of pride and arrogance these wings sometimes soar. One cannot help a feeling of grim satisfaction as one thinks of the termagant wife flourishing her domestic broom at the high flyer, and bidding him return to the common earth on the instant, or to beware of the consequences. The sudden meekness with which many a soaring eagle of a husband folds his wings and descends, is very edifying. Perhaps, after all, the affairs of the world, even of the married world, are not quite so badly arranged as some of our literary philosophers seem to think.

